# On the First Notice of a Re-Union after Entire Separation of Parts

**Published:** 1817-06

**Authors:** William Balfour


### *For the London Medical and Physical Journal.*

### *On the First Notice of a Re-union after entire Separation of Parts.* By WILLIAM BALFOUR, M.D.

Suum cuique.

I CONCEIVE it to be a duty I owe to myself and to some other practitioners, to correct a statement which appears in the Number of your Journal for this month, under the head *Medical and Philosophical Intelligence*, which is to the purport, that "At a recent sitting of the Society of Medicine, M. L'Espagnal communicated a very singular fact,"—the accidental amputation and re-union of the last phalanx of a finger; and, that this is the only "well-authenticated" case of re-union being accomplished after complete separation. Now, I deny that M. L'Espagnal's case is either "singular," or so "well-authenticated," as some others that are recorded in your own Journal, and those at no distant date. George Pedie's, which was published by me in the Edin. Med. and Surg. Journal for Oct. 1814, and afterwards noticed in your Journal, is the first well-authenticated case, in ancient or modern times, of the complete re-union of a piece of animal matter, consisting of flesh and bone, after entire separation from the living body.* Any case of the kind previously recorded, rests on the authority of the individual who reported it, and, of course, was treated with universal discredit, ridicule, and contempt. Pedie's case, on the contrary, besides being more important in itself, from being on a larger scale than any thing of the kind that ever occurred before, is attested by three persons on oath, and was witnessed by between twenty and thirty of his fellow workmen, all of them disinterested persons. To me, therefore, belongs the honor of having decided the question of the practicability of reuniting parts entirely separated from the living body. This claim, indeed, no one has attempted to resist. On the contrary, I have received congratulations on the subject, from various parts of Scotland and England, from France, Switzerland, the East and West Indies. M. L'Espagnal, therefore, is indebted to me for the fact he communicated to the Society of Medicine of Paris. That this is the case, is rendered probable, at least, from the consideration, that Pedie's case is mentioned every season as the first well-authenticated fact of the kind, by all the teachers of anatomy and surgery in

* This is acknowledged in the Transactions of the Royal Institute of France for 1815.

this this school of medicine, to which there is an afflux of students from every quarter of the world; that it was recorded in every periodical publication, and the subject of conversation and of admiration among all ranks of people in the United Kingdom; that it was inserted in the Bibliotheque de Medecine Britannique, and, therefore, must quickly have become as universally known in France and in Switzerland, as in Britain. Dr. Thomson, of London, gives the case of a servant girl in Switzerland, who accidentally cut a piece out of the fleshy part of one of her hands, and had it replaced by her mistress, a farmer's wife, with complete success.—This was done entirely on the faith of Pedie's case. "Such are the facts, (says Dr. Thomson,) respecting this curious and important subject, as far as I am acquainted with them. They seem amply sufficient to remove the scepticism of the most obstinate unbeliever; and, I have no doubt, that the practice will speedily become quite common. Dr. Balfour seems entitled to the whole merit of having called the attention of practitioners to this great process of nature, and to have demonstrated its practicability in the most satisfactory manner."—*Dr. Thomas Thomson's Annals of Philosophy, April* 1816.

M. Percy has reported Pedie's case, *textuellement*, and says, "J'avoue que je ne connaissais point d'examples bien averés de doigts rendu à la vie, et bien cicatrises après leur entière et complette séparation, lorsque, dernièrement, le journal où M. Balfour a consigné ceux qui lui sont propres, m'est tombé dans les mains."—*Dictionaire des Sciences Medicales, tome douzieme, au mot—Ente Animale.*

Having thus vindicated my claim "to the whole merit of having called the attention of practitioners to this great process of nature;" it remains I should do justice to those who quickly followed my example, and who, with that candour which is to be found in great minds only, frankly acknowledged their obligations to me for the brilliant success that attended their efforts.

Mr. Henry W. Bailey, surgeon, Thetford, Norfolk, communicated to me the case of a man who had suffered accidental amputation of the last phalanx of the middle finger, while supplying a chaff machine. The parts were separated nearly an hour and a half before being replaced, but complete re-union took place. This is surely "a very singular fact."

Mr. Thomas Hunter, surgeon, Port Glasgow, communicated to me the case of a man, who, with an axe, struck off his right thumb close by the root. Mr. Hunter replaced the amputated amputated part with complete success, and the man recovered the use of his thumb, the motion of the joint excepted. These cases were published in the Edinb. Med. and Surg. Journal for July and October 1815, and subsequently noticed in the London Med. and Phys. Journal. The knowledge of these facts must, therefore, be co-extensive with the circulation of these Journals. With what consistency and truth, then, can any person come forward, at this time of day, and assert, that "numerous cases have recently been quoted in the various Medical Journals of Europe, of examples of uniting by a suture, and, in some cases, by the first intention; but in, we believe, all these cases, the part was not entirely cut off, but hung by a particle of the skin?"

*Edinburgh; May* 10, 1817.

---

[We are concerned Dr. Balfour should feel so sensibly an invasion of a property which can only exist in "other's breath." We conceived that, the same publicity being afforded to all the facts, the brilliancy of Dr. B.'s must for ever remind our readers of its priority; and, on that account only, thought it unnecessary to add any remark at the end of our correspondent's communication. —EDIT.]

---

### COLLECTANEA MEDICA,

CONSISTING OF

